# A novel test for type-I allergy based on crosslink formation of immunoglobulin-E receptors by allergen-specific immunoglobulin-E antibodies and an allergen

**DOI:** 10.1038/s41598-023-46730-8

**Published:** 2023-11-11

**Authors:** Yuki Koga, Soichiro Ishii, Tomoharu Yokooji, Konomi Yamamoto, Ryohei Ogino, Takanori Taogoshi, Hiroaki Matsuo

**Affiliations:** 1 Department of Pharmaceutical Services, Graduate School of Biomedical and Health Sciences, Hiroshima University, 1-2-3 Kasumi, Minami-ku, Hiroshima, 734-8553 Japan; 2 Department of Frontier Science for Pharmacotherapy, Graduate School of Biomedical and Health Sciences, Hiroshima University, Hiroshima, Japan

**Keywords:** Immunological techniques, Diseases

## Abstract

Detection of allergen-specific immunoglobulin E (IgE) antibodies (Abs) in serum would allow for screening of the causative allergen in patients with type-I allergy. In this study, we developed a new assay method to detect allergen-specific IgE Abs, which involved crosslinking the plural FcεRIα molecules with an allergen and detection using an amplified luminescence proximity homogeneous assay (AlphaCL). First, the allergen concentration, bead concentrations, and incubation time were optimized for the detection of anti-2,4-dinitrophenyl (DNP) IgE Abs in buffer. Under optimal conditions, AlphaCL was able to detect DNP-specific IgE Abs in simulated human serum at levels comparable to those in serum from type-I allergic patients. When AlphaCL was used to detect anti-DNP IgE Abs, no signal counts were obtained with the monovalent allergen 2,4-dinitrophenylated poly-γ-glutamic acid, whereas high signal counts were obtained with the multivalent allergen DNP-BSA. This confirmed that AlphaCL could specifically detect allergen-specific IgE Abs with the ability to crosslink a multivalent allergen. In summary, we have established a new assay model using AlphaCL to detect allergen-specific IgE Abs with FcεRIα crosslinking ability in human serum. This simple and practical assay model may be applied as a new diagnostic tool for patients with type-I allergy.

## Introduction

Type-I allergic symptoms are elicited by crosslinking of plural allergen-specific immunoglobulin E (IgE) antibodies (Abs) bound to high-affinity IgE receptor alpha subunits (FcεRIα) on the surface of mast cells and basophils with an allergen^1,2^. The detection of allergen-specific IgE Abs in serum could be applied to screen for the causative allergen in patients with type-I allergy represented by food allergy^3–6^. In clinical practice, fluorescence enzyme immunoassays (FEIAs), such as ImmunoCAP™, are widely used to measure the serum levels of allergen-specific IgE Abs^7–9^. However, the FEIA system detects IgE Abs regardless of their ability to crosslink the plural FcεRIα with an allergen, resulting in false-positive diagnoses^10,11^. The histamine release test (HRT) and basophil activation test (BAT) using basophils from the peripheral blood of a patient are performed to detect IgE Abs with the ability to crosslink^12,13^. However, the differences in basophils condition after blood sampling can affect the results^14^. In addition, HRT and BAT cannot be applied to patients with basophils that are unreactive or show low reactivity to the anti-IgE Abs used as positive controls^15^. To improve these tests, the IgE crosslinking-induced luciferase expression (EXiLE) test using cultured mast cells was established for the detection of allergen-specific IgE Abs^16,17^. However, the EXiLE test is complicated in terms of cell management and manipulation, and a limited number of clinical facilities can perform this test. In the case of food allergy, a false-positive diagnosis may decrease the quality-of-life of a patient because of an unnecessary elimination diet, and a false-negative diagnosis could lead to anaphylaxis if the causative allergen in food is ingested^18–20^. Therefore, a highly sensitive and accurate assay method for the diagnosis of type-I allergy is needed in clinical practice.

Recently, we established a new assay, the amplified luminescence proximity homogeneous assay (AlphaCL), to detect functional autoantibodies against FcεRIα and IgE Abs, which can crosslink the plural FcεRIα molecules and IgE Abs on the surface of mast cells and basophils present in sera, in patients with autoimmune chronic spontaneous urticaria (aiCSU)^21^. Alpha technology is a bead-based proximity assay, which is used to detect the molecular interactions among biomolecules^22^.

**Figure 1:**
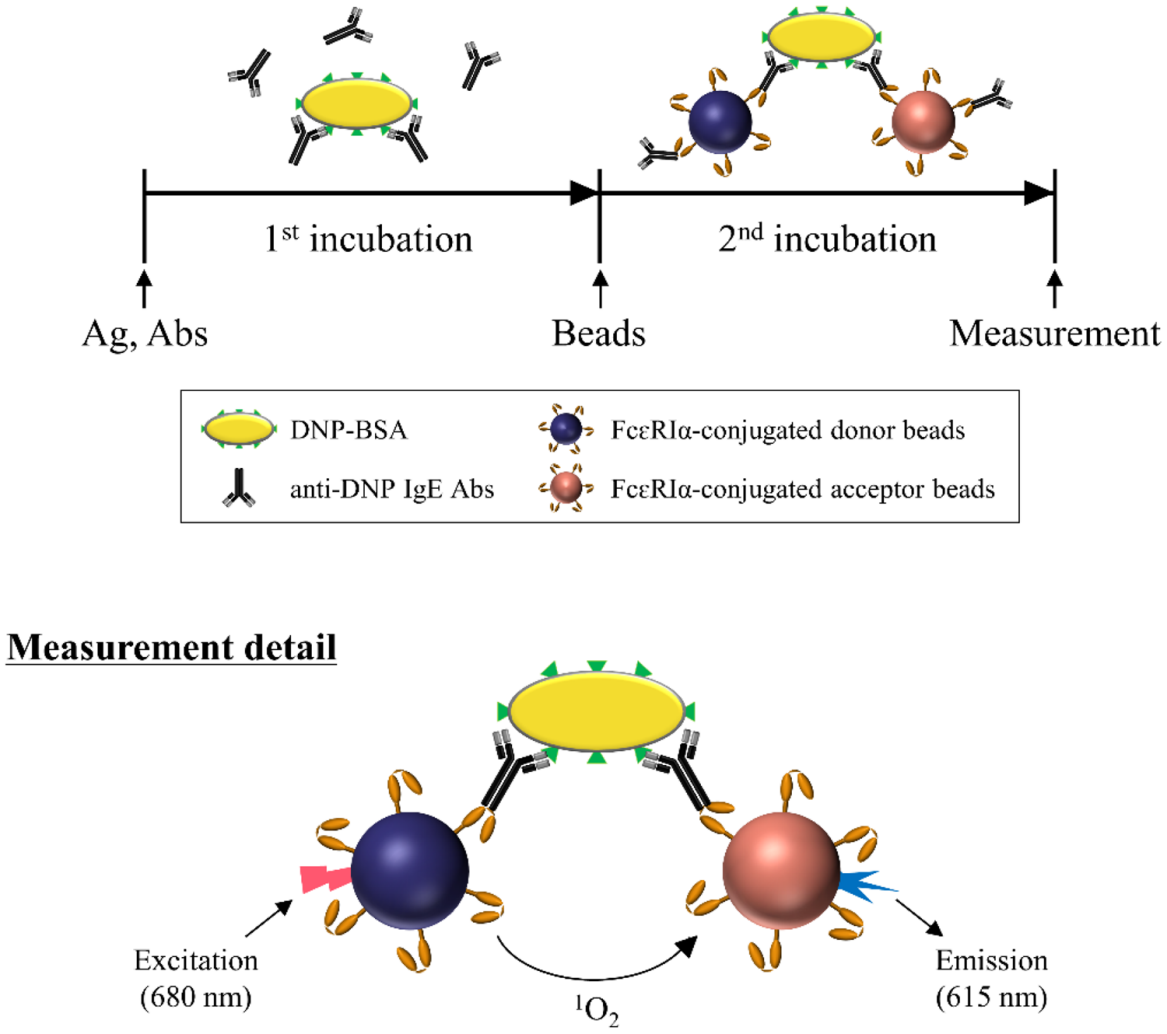
A schematic showing the principle of AlphaCL to detect anti-DNP IgE Abs. When each bead comes into proximity by crosslinking of the allergen-specific IgE Abs bound to FcεRIα conjugated on each bead with an allergen, the acceptor beads can receive the singlet oxygen that is released from the donor beads by excitation at 680 nm. The energy generated by singlet oxygen in the acceptor beads results in a sharp emission peak at 615 nm. DNP, 2,4-dinitrophenylated.

In the current study, we developed a new assay method based on AlphaCL to detect allergen-specific IgE Abs, which can crosslink the plural FcεRIα molecules with an allergen. The principle of this assay model imitates mast cell activation by crosslinking of allergen-specific IgE Abs bound to FcεRIα on the surface of the beads with an allergen. As shown in Fig. 1, we constructed the assay model using 2,4-dinitrophenylated albumin from bovine serum (DNP-BSA) as an allergen and mouse anti-DNP IgE Abs as allergen-specific IgE Abs. DNP-BSA is useful as an allergen model because (1) it has a molecular weight similar to common food allergens, (2) it is used as a typical allergen model with multiple epitopes, and (3) it is commercially available including purified anti-DNP IgE Abs. According to the certificate of analysis for DNP-BSA used in this study, ~ 30 DNP groups conjugated to one BSA molecule.

This new assay model differs from our previous ones in the process of cross-link formation between acceptor and donor beads. When detecting the functional IgG autoantibodies against IgE Abs and FcεRIα in sera from aiCSU patients, the autoantibodies bind directly to each protein conjugated on both beads and can cross-link beads to each other. In contrast, when detecting the functional IgE Abs specific to an allergen in sera from patients with allergy, the allergen-specific IgE Abs must bind to the FcεRIα protein on the beads. Therefore, tripartite complex must be formed between the allergen, the allergen-specific IgE Abs and the FcεRIα conjugated on beads. In addition, a higher sensitivity is required to detect allergen-specific IgE Abs than IgG autoantibodies because the amount of IgE Abs in the serum may be much lower than that of IgG Abs. Furthermore, it is important to consider monovalent allergens. In this study, we focused on these issues and established optimal detection model for allergen-specific IgE Abs.

## Results

### Determination of the optimal concentrations of DNP-BSA and acceptor/donor beads, and the optimal incubation time to detect anti-DNP IgE Abs

**Figure 2:**
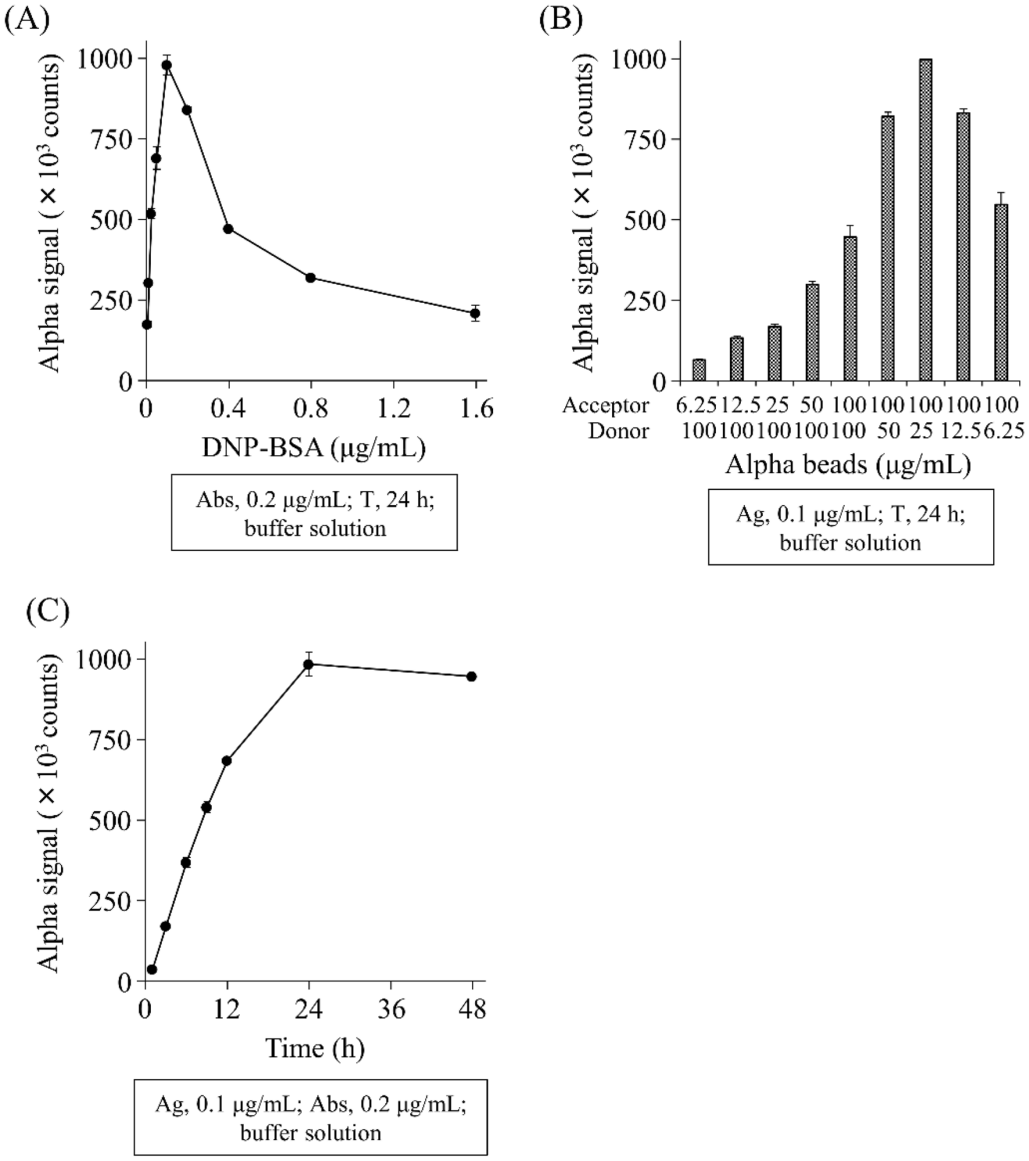
Determination of the optimal concentration of DNP-BSA (**A**) and acceptor/donor beads (**B**), and the optimal incubation time (**C**) for AlphaCL to detect anti-DNP IgE Abs. Each signal count was subtracted by that in the absence of anti-DNP IgE Abs. All experiments were performed in duplicate. Abs, anti-DNP IgE antibodies; T, incubation time; Ag, DNP-BSA.

To optimize the detection of anti-DNP IgE Abs in immunoassay buffer, we evaluated the appropriate concentrations of DNP-BSA and beads, and the incubation time. DNP-BSA gradually increased the signal counts up to 0.1 μg/mL, and then decreased the signal counts at concentrations of 0.00625–1.6 µg/mL (Fig. 2A). In addition, the signal counts increased by adding acceptor beads in a concentration-dependent manner and showed a peak count at concentrations of 100 µg/mL (acceptor) and 25 µg/mL (donor) for bead concentrations of 6.25–100 µg/mL (Fig. 2B). The signal counts increased gradually with time up to 24 h and then reached a plateau during 1–48 h incubation (Fig. 2C). For further studies, we detected anti-DNP IgE Abs using 0.1 μg/mL of DNP-BSA, 100 µg/mL of acceptor, and 25 µg/mL of donor beads over a 24 h incubation.

### The concentration-dependent crosslinking of acceptor and donor beads by anti-DNP IgE Abs in buffer or human serum

**Figure 3:**
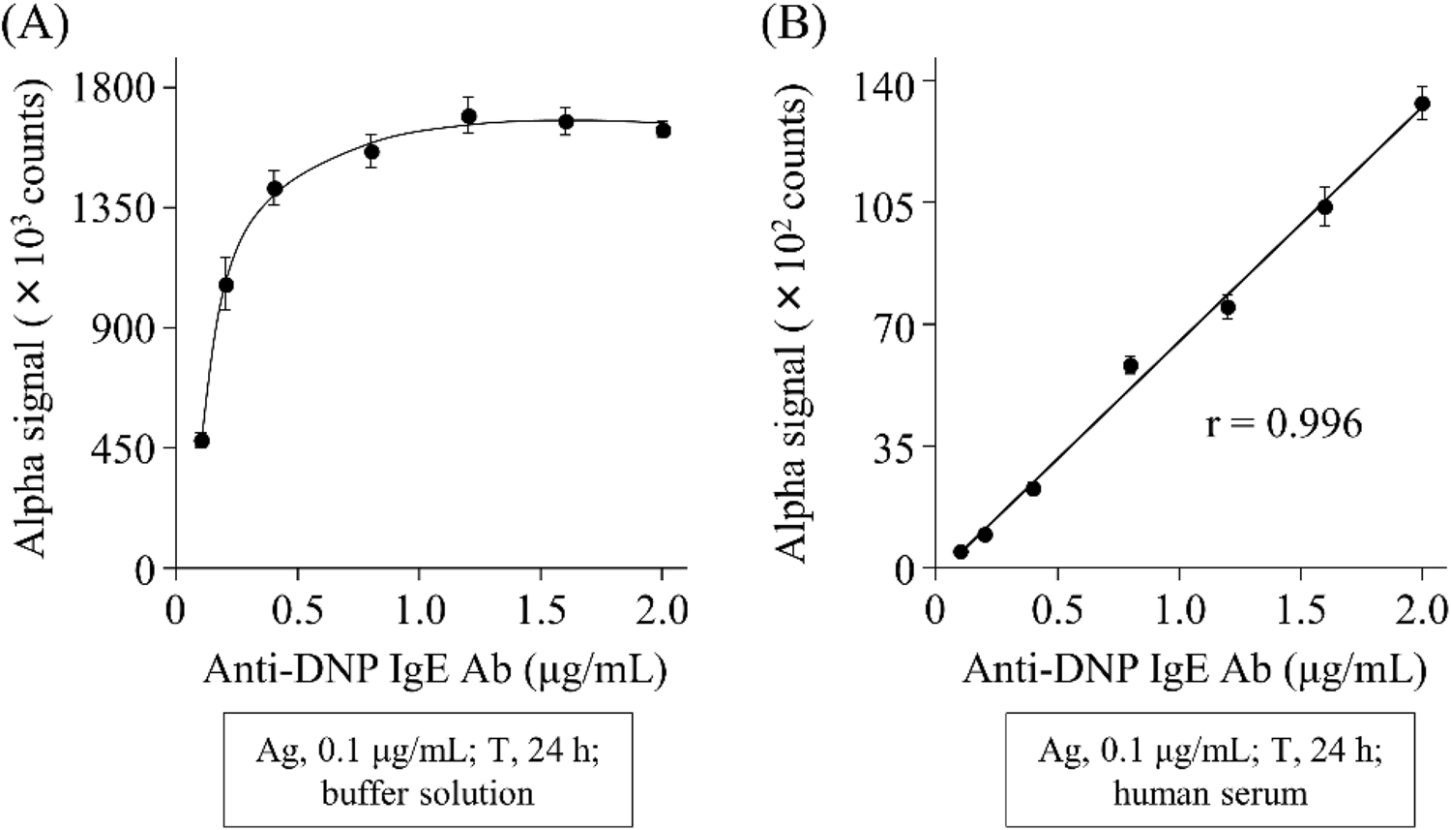
The concentration-dependent crosslinking of acceptor and donor beads by anti-DNP IgE Abs in buffer (**A**) or human serum (**B**) at concentrations of 0.2–2 µg/mL. Each signal count was subtracted by that in the absence of anti-DNP IgE Abs and the value represents the means ± S.D. of the mean. All experiments were performed in duplicate. Abs, anti-DNP IgE antibodies; T, incubation time; Ag, DNP-BSA.

To confirm whether AlphaCL can detect allergen-specific IgE Abs in sera from patients with type-I allergy, we attempted to detect DNP-specific IgE Abs in human serum. We prepared a simulated human serum from patients with type-I allergy by adding anti-DNP IgE Abs into commercially available human pooled serum. DNP-BSA was incubated with an equivalent volume of buffer or the simulated serum sample containing anti-DNP IgE Abs (0.1–2.0 µg/mL). In the buffer solution, the signal counts logarithmically increased depending on the concentrations of anti-DNP IgE Abs up to 0.8 μg/mL, and then reached a plateau (Fig. 3A). In the human serum, the signal counts were linearly increased by adding anti-DNP IgE Abs in a concentration-dependent manner (Fig. 3B).

### Differences in the detection of anti-DNP IgE Abs against monovalent allergen and multivalent allergen by ELISA and an AlphaCL assay

**Figure 4:**
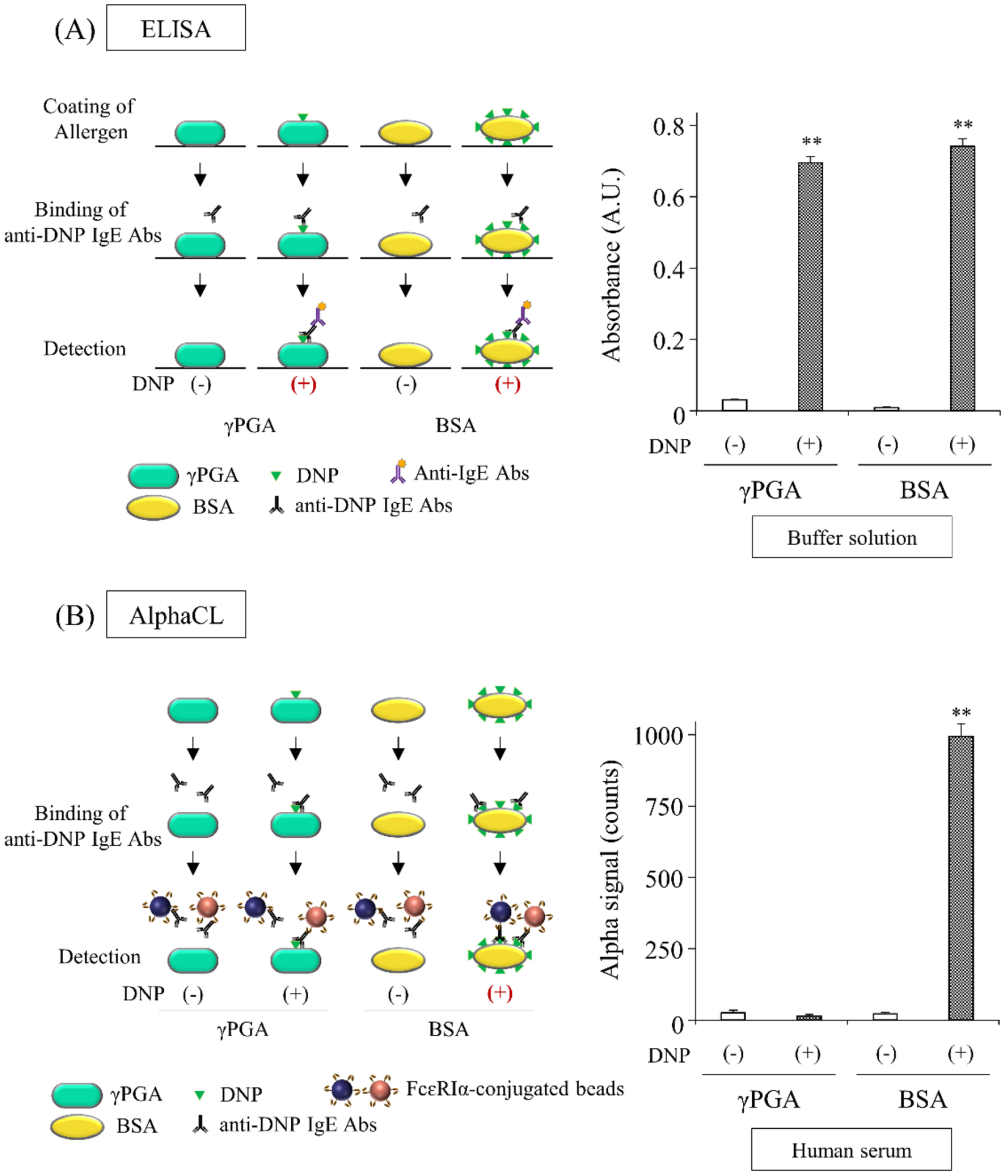
Differences in the detection of anti-DNP IgE Abs against monovalent allergen, DNP-γPGA, and multivalent allergen, DNP-BSA, by ELISA (**A**) and the AlphaCL assay (**B**). Each signal count was subtracted by that in the absence of anti-DNP IgE Abs and the value represents the means ± S.D. of the mean. All experiments were performed in duplicate. ^**^P < 0.01 was considered statistically significant between proteins conjugated with DNP group ( +) and unconjugated (–).

To evaluate whether AlphaCL can specifically detect the allergen-specific IgE Abs with the ability to crosslink a multivalent allergen, we attempted to compare the differences in the detection of anti-DNP IgE Abs against monovalent allergen and multivalent allergen by ELISA and an AlphaCL assay. We used 2,4-dinitrophenylated poly-γ-glutamic acid (DNP-γPGA) as a monovalent allergen. The absorbance values increased in the presence of DNP-γPGA or DNP-BSA compared with unconjugated γPGA or BSA (Fig. 4A). In the AlphaCL assay, no signal counts were obtained when unconjugated γPGA or BSA were mixed with beads and anti-DNP IgE Abs (Fig. 4B). Furthermore, no signal counts were obtained by adding DNP-γPGA, whereas high signal counts were obtained by adding DNP-BSA (Fig. 4B).

## Discussion

Allergen-specific IgE Ab detection can be utilized to screen for the causative allergen in patients with type-I allergy^3–9,23–26^. However, conventional assays have low specificity and involve complicated procedures to detect the IgE Abs that crosslink plural FcεRI on the surface of mast cells and basophils. In this study, we developed a new assay method using AlphaCL to detect allergen-specific IgE Abs, which can crosslink the plural FcεRIα molecules with an allergen. Our new assay model provides the following five benefits compared with conventional assays: (1) the use of non-basophil cells or cultured mast cells, (2) the highly specific detection of functional allergen-specific IgE Abs, (3) the feasibility of the assay in any facility, (4) the minimally invasive nature of the assay for subjects, and (5) the fact that it is substitutable for various allergens.

To optimize the detection for anti-DNP IgE Abs, we first determined the appropriate concentrations of DNP-BSA and beads, and the incubation time. DNP-BSA increased the signal count gradually up to 0.1 μg/mL and then decreased (Fig. 2A). The decreased signal under high concentration of DNP-BSA may be explained as follows: (1) the signal is gained because one molecule of DNP-BSA can bind several crosslinked anti-DNP IgE Abs, (2) as the concentration of DNP-BSA increases, the amounts of anti-DNP IgE Abs are not enough for those of DNP-BSA. Signal counts for bead concentrations peaked at 100 µg/mL for acceptor and 25 µg/mL for donor beads (Fig. 2B). This may indicate that the donor beads conjugate with four times as many FcεRIα molecules as acceptor beads although we could not confirm the amounts of FcεRIα molecule conjugated on each bead. The 24 h incubation showed the peak signal counts and then reached a plateau (Fig. 2C), suggesting that the optimal incubation time is 24 h in this study. The long incubation time might be a disadvantage for use as a diagnostic tool. To shorten the incubation time, we attempted to change the assay procedure to complexing anti-DNP IgE Abs with beads first ant then adding DNP-BSA in a preliminary study. However, the total incubation time to detection did not change. Alternatively, this assay can be performed in a shorter time than 24 h if the increased sensitivity of the assay results in significantly higher signal counts in patients than in healthy subjects in a short time.

Next, we detected DNP-specific IgE Abs in simulated human serum using AlphaCL. The signal counts in the buffer increased up to 0.8 μg/mL of anti-DNP IgE Abs, and then reached a plateau (Fig. 3A). This result indicates that the binding sites of anti-DNP IgE Abs on 0.1 μg/mL of DNP-BSA were saturated at concentrations of ≥ 0.8 μg/mL. On the other hand, the signal counts in the simulated serum were linearly increased in a concentration-dependent manner of anti-DNP IgE Abs (Fig. 3B). However, the signal counts in serum were < 1% of those in the buffer at the same anti-DNP IgE Ab concentration. The detection limit of anti-DNP IgE Abs in the presence of human serum using AlphaCL was estimated as 0.1 μg/mL. The reason for the remarkable decrease in signal count following the addition of human serum is unclear. The binding of allergen-specific IgE Abs to FcεRIα proteins on the surfaces of beads might be disrupted by the highly abundant non-specific free IgE Abs in serum. Mogues et al. reported that human serum contains the anti-BSA IgG Abs^27^. Therefore, high concentration of anti-BSA IgG Abs in human serum might disrupt the bindings of anti-DNP IgE Abs with DNP-BSA in this assay model. In addition, we speculate that the detection of anti-DNP IgE Abs may be disrupted by the highly abundant serum components such as non-specific free IgE and IgG Abs as well as anti-BSA IgG Abs. In addition, the serum components with free-radical scavenging capacity may inhibit the transfer of singlet oxygen from the donor beads to the acceptor beads^28^. The concentrations of allergen-specific IgE Abs were reported to be ~ 0.00084–0.24 μg/mL in sera from patients with type-I allergy^29^. Therefore, it may be necessary to remove the interfering components including IgG Abs in the serum to improve the sensitivity of our assay.

Next, we demonstrated that AlphaCL could specifically detect the allergen-specific IgE Abs with the ability to crosslink an allergen with multivalent epitopes using DNP-γPGA as an allergen with a monovalent epitope. DNP group can conjugate to amino group in a protein molecule. BSA has 59 lysine residues and one amino acid at the N-terminus in one molecule^30^. As mentioned above, ~ 30 DNP groups conjugated to one BSA molecule used in this study. In contrast, one γPGA molecule can be conjugated with only one DNP group because this protein has one amino group at the N-terminus. The higher absorbance values in ELISA were obtained in the presence of DNP groups compared to their absence (Fig. 4A). This result indicates that ELISA detects anti-DNP IgE Abs that bind to proteins conjugated with one or more DNP groups, i.e., ELISA detects anti-DNP IgE Abs regardless of the ability to crosslink the plural FcεRIα with an allergen. In contrast, no signal counts were obtained by adding DNP-γPGA, whereas high signal counts were obtained by adding DNP-BSA in AlphaCL assay (Fig. 4B). These results indicate that AlphaCL can specifically detect allergen-specific IgE Abs with the ability to crosslink the plural FcεRIα with a multivalent allergen. Therefore, AlphaCL may decrease the occurrence of false-positive results observed with FEIAs.

The limitation of this study was that we used a simulated serum sample prepared from pooled healthy subjects instead of serum from patients with type-I allergy. Previous reports have shown that patients with type-I allergy possess allergen-specific IgG Abs, in addition to allergen-specific IgE Abs^1,31,32^. These allergen-specific IgG Abs may interfere the IgE Ab binding to an allergen, resulting in low sensitivity in our assay. Furthermore, the AlphaCL needs 24 h incubation to detect functional allergen-specific IgE compared to that in conventional diagnostic tools including HRT and BAT. Further studies are necessary to obtain the result rapidly. We used mouse anti-DNP IgE Abs in this study, as IgE Ab-binding to human FcεRIα proteins was confirmed by ELISA in our preliminary study (data not shown). Further studies are necessary to confirm whether the levels of allergen-specific IgE Abs in sera from patients with type-I allergy can be determined by AlphaCL.

In conclusion, we established a new assay method using AlphaCL to detect allergen-specific IgE Abs with FcεRIα crosslinking ability in serum. This simple and practical assay method could be applied to detect various allergen-specific IgE Abs and has potential as a new diagnostic tool for patients with type-I allergy.

## Methods

### Determination of the optimal concentration of DNP-BSA and acceptor/donor beads, and the optimal incubation time to detect anti-DNP IgE Abs

To construct our new assay model, we used DNP-BSA (Sigma-Aldrich, St. Louis, MO, USA) as an allergen and mouse anti-DNP IgE Abs (1 mg/mL, YAMASA, Chiba, Japan) as allergen-specific IgE Abs. When donor beads come into proximity by the crosslinking of allergen-specific IgE Abs bound to FcεRIα conjugated on the beads with an allergen, the acceptor beads can receive the singlet oxygen that is released from the donor beads by excitation at 680 nm. The energy generated by singlet oxygen in the acceptor beads results in a sharp emission peak at 615 nm (Fig. 1)^22^. The levels of allergen-specific IgE Abs can be quantitatively determined by measuring the intensity of emission peak signals using a multimode plate reader.

To detect anti-DNP IgE Abs, we also prepared donor and acceptor beads (each 5 mg/mL, PerkinElmer, Waltham, MA, USA) conjugated with FcεRIα proteins (R&D SYSTEMS, Minneapolis, MN, USA) according to the manufacturer’s protocol (PerkinElmer). The beads were diluted with immunoassay buffer (PerkinElmer) and mixed at appropriate concentrations. To optimize anti-DNP IgE Ab detection, we evaluated the allergen and bead concentrations, and the incubation time. To determine the DNP-BSA concentration, 20 µL of DNP-BSA (0.00625–1.6 µg/mL) and 20 µL of anti-DNP IgE Abs (0.2 µg/mL) were added to a 1/2 AreaPlate-96 plate (PerkinElmer) and incubated for 1 h at room temperature (RT). Then, 10 µL of the bead mixture (acceptor, 100 µg/mL; donor, 25 µg/mL) was incubated in the same plate for 24 h at RT in the dark. Our previous report showed that 24 h incubation was optimal to detect the anti-IgE autoantibodies^21^. Based on this finding, we first set the incubation time to 24 h to determine the optimal concentration of DNP-BSA. Then, we also determined the optimal bead concentrations and incubation time at the optimal concentration of DNP-BSA (0.1 µg/mL). For evaluation of the bead concentrations, 20 µL of DNP-BSA (0.1 µg/mL) and 20 µL of anti-DNP IgE Abs (0.2 µg/mL) were incubated in the plate for 1 h at RT. Then, 10 µL of the bead mixture at different concentrations (concentration range, 6.25–100 µg/mL) were added and incubated in the same plate for 24 h at RT in the dark. For the incubation time, 20 µL of DNP-BSA (0.1 µg/mL) and 20 µL of anti-DNP IgE Abs (0.2 µg/mL) were incubated in the plate for 1 h at RT. Then, 10 µL of the bead mixture (acceptor, 100 µg/mL; donor 25 µg/mL) were incubated in the same plate for 1–48 h at RT in the dark. The Alpha signal counts were measured using a multimode plate reader (NIVO™, PerkinElmer), and calculated as net counts by subtracting the counts in buffer containing no anti-DNP IgE Abs from the total counts in each sample. All experiments were performed in duplicate.

### The concentration-dependent crosslinking of acceptor and donor beads by anti-DNP IgE Abs in buffer or human serum

To detect DNP-specific IgE Abs in human serum using AlphaCL, we prepared simulated human serum by adding anti-DNP IgE Abs into commercially available human pooled serum (Sigma-Aldrich). DNP-BSA (0.1 µg/mL) was incubated with an equivalent volume of buffer or the simulated serum sample containing anti-DNP IgE Abs at concentrations of 0.1–2.0 µg/mL for 1 h, and then 10 µL of the bead mixture (acceptor, 100 µg/mL; donor 25 µg/mL) was incubated in the plate for 24 h. The signal counts were measured and calculated according to the method described above. All experiments were performed in duplicate.

### Differences in the detection of anti-DNP IgE Abs against monovalent allergen and multivalent allergen by ELISA and AlphaCL assays

To demonstrate that AlphaCL can only detect allergen-specific IgE Abs to multivalent allergens with at least two or more IgE-binding sites, we prepared DNP-γPGA as a monovalent allergen, according to the method described by Sanger et al.^33^ with a slight modification. Briefly, 1-fluoro-2,4-dinitrobenzene (50 µL, Nacalai Tesque, Kyoto, Japan) dissolved in 500 µL of ethanol was slowly mixed with γPGA sodium salt (50 mg, Wako, Osaka, Japan) dissolved in 500 µL of 1.2 M sodium hydrogen carbonate solution. After incubation for 2 h at RT, the solution was centrifuged at 3000×*g* for 30 min using Centricut® Mini V-10 (KURABO, Osaka, Japan). Then, the pellet was collected by dissolving with ultrapure water and acidified to pH 7.0 with diluted hydrochloric acid.

ELISA was performed according to the method described by Yamada et al.^34^ with a slight modification. Briefly, F8 MaxiSorp loose Nunc-Immuno Modules (Thermo Fisher Scientific, Waltham, MA, USA) were coated with 100 μL of DNP-conjugated or unconjugated γPGA (1 mg/mL), or BSA (10 µg/mL, Sigma-Aldrich), dissolved in phosphate-buffered saline (PBS, pH 7.4) overnight at 4 °C. After washing with PBS containing 0.05% Tween-20 (PBS-T) three times, the plates were incubated with 1% Block Ace® (DS Pharma Biomedical, Osaka, Japan) for 2 h at 37 °C. After washing with PBS-T, 100 μL of anti-DNP IgE Abs dissolved in 1% Block Ace® was applied and incubated for 2 h at 37 °C. The wells were washed with PBS-T, and then incubated with 100 μL of horseradish peroxidase-conjugated goat anti-mouse IgE Abs (Bio Trend, Köln, Germany) (diluted 1:7000 with 1% Block Ace® solution) for 1 h at RT. The wells were washed with PBS-T and incubated with 100 μL of 3,3′,5,5′-tetramethylbenzidine solution (KPL, Gaithersburg, MD, USA) at RT. After a 15-min incubation, the reaction was terminated with 100 μL of 1 M phosphoric acid. Absorbance was determined at 450 nm against 620 nm as a reference using a Multiskan GO™ (Thermo Fisher Scientific).

For the AlphaCL assay, DNP-conjugated or unconjugated γPGA (10 µg/mL, 20 µL) or BSA (0.1 µg/mL, 20 µL) was incubated with 20 µL of the simulated serum sample containing anti-DNP IgE Abs (0.2 µg/mL) in the plate for 1 h at RT. Then, 10 µL of the bead mixture (acceptor, 100 µg/mL; donor 25 µg/mL) was incubated in the plate for 24 h at RT in the dark. Measurements were performed using NIVO™. The signal counts were measured and calculated according to the method described above. All experiments were performed in duplicate.

### Statistics

Data were displayed as the means ± standard deviations of the mean (S.D.), and the strength of the correlation in the results between anti-DNP IgE Abs concentration and Alpha signals were analyzed by Spearman’s correlation coefficient by rank test. Mean value differences between DNP-labeled and unlabeled protein were assessed using Student’s t-tests. *P* < 0.05 was considered statistically significant.

## Data Availability

All data analyzed/generated during this study are included in this article.
